# Identification of genes controlling metastatic behaviour.

**DOI:** 10.1038/bjc.1991.4

**Published:** 1991-01

**Authors:** I. R. Hart, D. Easty


 © Macmillan Press Ltd., 1991

GUEST EDITORIAL

### Identification of genes controlling metastatic behaviour

I.R. Hart & D. Easty

*Imperial Cancer Research Fund Laboratories, Lincoln's Inn Fields, London WC2A 3PX, UK.*

The obvious importance of metastatic spread in determining clinical outcome in cancer patients has fuelled efforts to increase understanding of the pathobiology of this process in the hope that better understanding eventually can be translated into improved therapy. Considerable impetus was given to the experimental analysis of the mechanisms underlying tumour dissemination by the demonstration that populations of metastatic and non-metastatic cells could pre-exist within the same parental tumour (Fidler & Kripke, 1977). Selection during the complex, sequential events of cancer spread might ensure that the cellular composition of secondary tumours differed from that of primary tumours (Poste & Fidler, 1980). The opportunity was thus provided to define those characteristics of importance in regulating metastatic spread by comparing primary and secondary growths or by comparing variant lines differing only in their malignant behaviour (Hart *et al.*, 1989). However, recent results with genetically tagged cells have shown that, in certain tumour types, overgrowth of the primary tumour with metastatic cell populations can be a particularly rapid event (Kerbel *et al.*, 1988). Therefore, the identification of differences between local and distant growths may not be feasible in all instances. Nonetheless recent efforts to identify and isolate specific genes controlling metastatic behaviour often have utilised material derived from primary and secondary growths. Alternatively closely related variant lines with different metastatic potential frequently have provided the starting material used in these studies. These recent approaches to the direct identification and isolation of genes determining the metastatic phenotype form the subjects of this review.

### Transfection studies

Conversion of non-metastatic tumour cells to the fully metastatic phenotype has been achieved in a number of experimental tumour systems by the relatively simple expedient of transfecting the benign population with an activated oncogene, generally of the *ras* family (e.g. Kyprianou & Isaacs, 1990; Treiger & Isaacs, 1988). The process of metastasis has been considered to be a complex phenomenon developing late in tumour progression where accumulation of somatic mutations, coupled with strong environmental selection pressures, has led to the emergence of more aggressive clonal subpopulations (Nowell, 1976). Regulation of the process has been presumed to result from the activation/repression of a number of specific genes (Nicolson, 1988). The conversion of a benign to a malignant cell by a single gene may be explainable if the cells already express all other gene products necessary for metastasis; the exogenous gene simply completes the requisite repertoire. More surprising is that numerous groups have documented the fact that non-tumourigenic fibroblast cell lines acquire metastatic activity following transfection with either *ras* or protein kinase oncogenes (Bradley *et al.*, 1986; Egan *et al.*, 1987*a,b*; Greig *et al.*, 1985).

Received 16 July 1990; and in revised form 7 September 1990.

Though both N1H3T3 and 10T1/2 lines used in these studies are non-tumourigenic, a characteristic that is not always steadfastly maintained (Greig *et al.*, 1985), they have been grown in tissue culture for extended periods of time. The lines therefore may have accrued sufficient somatic mutations to require only the addition of the benefits conferred by the introduced gene to 'tip them over the edge' to full malignancy; in certain instances this movement to malignancy may be independent of the addition of any gene (Greig *et al.*, 1985). A somewhat similar picture is often apparent in clinical situations. For example, while *ras* mutations frequently occur at an early stage of development in certain human cancers, it is the total accumulation, rather than the order, of genetic alterations which appears to be the most important determinant underlying tumour progression (Fearon & Vogelstein, 1990). Less easy to understand in these terms is the report that early passage embryo cells, presumably relatively free of acquired mutations, can be rendered fully metastatic by transfection with the *ras* oncogene alone (Muschel *et al.*, 1985). It could be that in this instance introduction of the *ras* gene acted to increase genetic instability, thus accelerating the process of mutation acquisition, or that it acted as a fundamental control gene, possibly to regulate transcription of other genes involved in the metastatic process (Liotta, 1988). Interestingly therefore *ras* transfection has been accompanied by elevated expression of various proteases; enzymes which have often been implicated in the invasive process (Collier *et al.*, 1988; Denhardt *et al.*, 1987; Garbisa *et al.*, 1987).

Given that a single oncogene has resulted in acquisition of the full malignant phenotype it is not surprising that efforts have been made to isolate novel metastasis genes by the transfection of high molecular weight DNA from malignant cells into non-metastatic cells. As far as we are aware no gene, other than an activated *ras* oncogene (Thorgeirsson *et al.*, 1985; Ananthaswamy *et al.*, 1988, 1989), has yet been isolated and identified by this approach, although changes in metastatic behaviour have occurred as a consequence of transfer of genomic DNA (Radler-Pohl *et al.*, 1988).

### Differential screening of cDNA libraries

A number of assumptions underlie the use of transfection assays to detect genes conferring metastatic behaviour, not the least of which is that recipient, non-metastatic cells require a single, dominant gene for conversion to malignancy. However in the last year or so considerable attention has focused upon the possibility that genetic control of metastasis may be exerted via the deactivation of metastasis suppressor genes (Kerbel, 1989; Sobel, 1990) and such genes would not be detected in the transfection assays. For example Khokha and her colleagues (Khokha *et al.*, 1989) have shown that transfection of Swiss 3T3 cells with antisense RNA to Tissue Inhibitor of Metalloproteinases (TIMP) results in the gain of full metastatic capacity. Since an inverse correlation exists between production of the 28.5 kDa TIMP glycoprotein and the invasive capacity of transformed lines (Hicks *et al.*, 1984) is it feasible that the TIMP gene is acting as a suppressor of disseminative ability? Inactivation of the TIMP gene product by mutation/loss, or by the artificial introduction of antisense mRNA, could alter the normal balance between protease and protease inhibitor in the cell and thus lead to invasive and metastatic behaviour.

One way of detecting novel genes which may modulate invasion and metastasis that does not suffer from the necessity to score for a dominant trait is to utilise differential screening of cDNA libraries. The objective here is to detect cDNA clones corresponding to mRNAs which differ in abundance between related cell populations. No assumption is made as to the nature of the sought gene. The sensitivity of the differential screening technique is limited to detection of mRNA transcripts constituting $>0.01\%$ of the mRNA population; preliminary subtractive hybridisation raises this sensitivity to allow detection of transcripts constituting $>0.001\%$ of the mRNA (Hedrick *et al.*, 1984). A number of investigators have used this approach (see below) utilising mRNA prepared from established cell lines of metastatic and non-metastatic behaviour. Should primary and secondary tumours actually be composed of different populations of neoplastic cells then the approach should be equally feasible utilising fresh tumour tissues. Indeed Elvin *et al.* (1988) were able to use such starting material to isolate cDNA clones representing mRNAs differentially expressed during progression and metastasis of colorectal cancer. One possible complication of this approach is the potentially different degree of inflammatory cell infiltrate or tumour stroma between tumours at different sites. Differential mRNA expression simply might reflect cellular heterogeneity as a consequence of tumour location. Additionally cellular composition could vary to a marked extent within the tumour mass; thus the origin of the starting material could have a profound effect on subsequent results. These problems are avoided by the utilisation of pure cell lines.

To date the relatively small number of published studies which have used this technique have identified a mixture of novel and previously known genes. Thus Elvin *et al.* (1988) isolated pLM59, following screening of a small library of 5,000 colonies, a cDNA coding for an 0.8 kb RNA transcript which was more abundant in a liver metastasis than in either normal or primary tumour material and which was expressed preferentially in metastatic murine cell lines. The identified sequence subsequently was shown to encode an acidic ribosomal phosphoprotein, P2 (Sharp *et al.*, 1990) which plays a role in protein translation (Rich & Steitz, 1987). Similarly pGM21, a gene associated with high metastatic potential in rat mammary adenocarcinomas, identified after a subtractive hybridisation procedure, seems to be partially homologous to human elongation factor 1 subunit $\alpha$, which is involved in protein synthesis (Phillips *et al.*, 1990). In both these instances metastatic capacity is associated with enhanced expression of the identified gene; an enhanced expression that possibly relates more to the metabolic activity, and perhaps the growth characteristics, of the lines being analysed than to the obvious biochemical requirements of metastatic cells. Rather more understandable is the up-regulation of transin mRNA in squamous cell carcinomas as compared to non-invasive papillomas (Matrisian *et al.*, 1986). This is because the metalloprotease encoded for by the transin gene, which seems to be homologous to stromelysin, may play a major role in matrix degradation. Increased expression in metastatic cells of another gene, *mts*-1, has been described recently (Ebralidze *et al.*, 1989). In this study the phenol-emulsion reassociation technique (PERT) (Kohne *et al.*, 1977) was used to enrich for cDNA molecules specific for metastatic cells. The amino acid sequence deduced from the nucleotide sequence of the *mts*-1 gene was identical to the mouse $Ca^{2+}$ binding protein (Ebralidze *et al.*, 1989; Tulchinsky *et al.*, 1990). Assignment of the *mts*-1 gene to the $Ca^{2+}$ binding protein family gives little indication of its possible role in tumour dissemination. However, it has long been postulated that a defect in calcium binding activity might lead to a loss in cell-cell adhesiveness that would facilitate cell shedding from malignant tumours (Coman, 1944). Since some of the cell-cell adhesion molecules, such as L-CAM and N-cadherin, are calcium dependent it may be possible that cellular dissociation in malignancy occurs because of perturbations in the activity of these molecules as a consequence of the *mts*-1 gene product acting as a calcium sink.

To underline the fact that the metastatic phenotype may be a consequence both of increased expression of certain genes or decreased expression of others, other genes isolated by differential hybridisation protocols have been downregulated in expression in metastatic variants. Thus Schalken *et al.* (1988) found that fibronectin mRNA was down-modulated in metastatic variants of a rat prostate tumour. Similarly, using a rat mammary adenocarcinoma, a family of RNA transcripts encoded by the gene WDNM1 have been detected which are expressed at a 20-fold higher level in non-metastatic cell lines (Dear *et al.*, 1988). Application of the subtractive hybridisation techniques to the same system resulted in the identification of a second gene, WDNM2, encoding 1.7 kb mRNA, corresponding to a 28–30 kDa protein, which was regulated transcriptionally in a positive correlation with the non-metastatic phenotype (Dear *et al.*, 1989). No light has been shed on the possible role that WDNM2 has in the malignant process with the recent notification that a full length cDNA sequence of the WDNM2 gene exhibited complete homology to the rat NAD(P)H:menadione oxidoreductase cDNA (Dear, 1990).

Currently the most interesting and the most exciting gene implicated in regulating metastasis which has been identified by the differential hybridisation procedure is the nm23 gene identified by Steeg and co-workers (Steeg *et al.*, 1988). Originally identified and isolated from variant lines of the K1735 murine melanoma the human homologue of this gene has now been cloned (Steeg & Liotta, 1990). Expression of nm23 mRNA has been shown to be down-regulated in metastatic variants in a wide variety of experimental tumour models. Much more interesting than the results from even these well characterised rodent models is the demonstration of an association between lack of expression and high metastatic potential in human infiltrating ductal breast carcinomas (Bevilacqua *et al.*, 1989). Antibodies raised against peptides corresponding to the N-terminus of the predicted protein product of the nm23 gene have now been used to show that the situation at the protein level mirrors that occurring at the mRNA level (Steeg & Liotta, 1990). It seems that nm23 is perhaps the best candidate for a metastasis suppressor gene yet identified. Analysis by Southern blot of matched normal and tumour samples, from patients with lung and renal carcinomas, for restriction fragment length polymorphism has revealed loss of heterozygosity in 40–60% of cases, consistent with a suppressor type function (Steet & Liotta, 1990). Transfection of the nm23 gene, under the control of various promoters, back into metastatic lines results in loss of both experimental and spontaneous metastatic activity in those lines with high level expression of the transfected gene and seems to confirm the suppressor function of nm23 (Steeg & Liotta, 1990). This type of experiment would seem to be an absolute requirement to verify that candidate genes are indeed metastasis suppressor genes. Care, however, must be taken in both the performance of such experiments and in the interpretation of results accruing from them. Assessment of metastatic capacity solely by the monitoring of experimental metastasis, where tumour cells are injected directly into the bloodstream and resultant colonies are scored in the target organ at a later date, may simply identify tumour suppressor genes rather than specific metastasis suppressor genes. It is imperative therefore that measurement of the tumourigenicity of the various transfectants be included in the assay; either by separate injection or by using the spontaneous metastasis assay utilised by Steeg and Liotta (1990). The demonstration of 78% identity between the predicted amino acid sequence of the nm23 gene and the *Drosophila* abnormal wing disc (awd) gene (Rosengard *et al.*, 1989) raises intriguing questions about the role of this highly conserved gene in the processes of cellular differentiation and morphological pattern formation. Processes which mimic the aberrant situation operating in tumour dissemination. As if to emphasise the conservation of nm23 throughout the animal kingdom it has recently become apparent that there is striking homology between nm23 and a nucleoside diphosphate (NDP) kinase isolated from *Dictyostelium* (Wallet *et al.*, 1990). It may well be that this conservation will facilitate the understanding of the biochemical basis of nm23 gene activity.

### Summary and conclusions

Gain or loss of a wide variety of different gene products has been documented during the progression of various cancers (Klein & Klein, 1985). Many of these alterations have been detected initially by variation in reactivity to monoclonal antibodies in tissues isolated from discrete stages of tumour development. Subsequently the genes coding for the antigens recognised by these antibodies have been cloned and sequenced. Thus in malignant melanoma, for example, increased expression of the cell adhesion molecule ICAM-1 has been associated with an increased risk of metastasis (Johnson *et al.*, 1989). A similar correlation between enhanced expression and late stage disease in melanoma has also been reported for the MUC 18 antigen, which shows sequence similarity to the neural cell adhesion molecules of the immunoglobulin superfamily (Lehmann *et al.*, 1989). Somewhat in contrast to these results in malignant melanomas, where increasing malignancy appears to be correlated with increased expression of cell-cell adhesion molecules, are the findings from colorectal cancer where allelic deletions involving chromosome 18q, which tend to occur relatively late in progression, have been determined to involve a gene specifying a protein with sequence similarity to neural cell adhesion molecules (Fearon *et al.*, 1990).

Whether these changes in genes encoding cell-cell adhesion molecules are a necessary step in the development of metastatic capacity or whether they are 'accessory' changes (Klein & Klein, 1985) remains to be determined. Most probably, like described changes in such molecules as class I antigens or integrin receptors, they play an important though not necessarily deterministic role in modulating metastatic behaviour. The direct approach to identifying genes which are able to confer the metastatic phenotype has been by utilisation of transfection assays. Results from such assays have shown clearly that some, though not all, of the already identified oncogenes are capable of inducing the requisite changes in tumourigenic, but benign cells. More surprisingly, given the view that metastasis is a multistep phenomenon, these same genes have been found to have the capacity to convert non-tumourigenic fibroblasts directly to the fully malignant phenotype. A finding which raises interesting questions about metastatic capacity arising late in tumour progression. While the use of high molecular weight genomic DNA has brought similar malignant conversion no novel gene has, as yet, been characterised from such studies.

Differential hybridisation of cDNA libraries, which would not discriminate between accessory or deterministic genes, has identified a series of both known and novel genes expressed to different degrees in variants of differing metastatic capacity of which one, nm23, appears to be the most likely candidate for a metastasis-regulating gene. It is likely that these types of studies will continue in the future and will result in the isolation of interesting candidate genes. Analysis of these genes both *in vitro* and in transgenic mice (Hanahan, 1988) will offer additional insights into the genetic regulation of tumour metastasis, with the promise that such insights may lead to new treatment strategies aimed at protein products specifically involved in tumour dissemination.

### References

ANANTHASWAMY, H.N., PRICE, J.E., GOLDBERG, L.H. & BALES, E.S. (1988). Simultaneous transfer of tumorigenic and metastatic phenotypes by transfection with genomic DNA from a human cutaneous squamous cell carcinoma. *J. Cell. Biochem.*, **36**, 137.

ANANTHASWAMY, H.H., PRICE, J.E., TAINSKY, M.A., GOLDBERG, L.H. & BALES, E.S. (1989). Correlation between Ha-ras gene amplification and spontaneous matastasis in N1H 3T3 cells transfected with genomic DNA from human skin cancers. *Clin. Exp. Metastasis*, **7**, 301.

BEVILACQUA, G., SOBEL, M.E., LIOTTA, L.A. & STEEG, P.S. (1989). Association of low nm23 RNA levels in human primary infiltrating ductal breast carcinomas with lymph node involvement and other histopathological indicators of high metastatic potential. *Cancer Res.*, **49**, 5185.

BRADLEY, M.O., KRAYNAK, A.R., STORER, R.D. & GIBBS, J.B. (1986). Experimental metastasis in nude mice of N1H3T3 cells containing various ras genes. *Proc. Natl Acad. Sci. USA*, **83**, 5277.

COLLIER, I.E., WILHELM, S.M., EISEN, A.Z. & 6 others (1988). H-*ras* oncogene-transformed human bronchial epithelial cells (TBE-1) secrete a single metalloprotease capable of degrading basement membrane collagen. *J. Biol. Chem.*, **263**, 6579.

COMAN, D.R., (1944). Decreased mutual adhesiveness. A property of cells from squamous cell carcinomas. *Cancer Res.*, **4**, 625.

DEAR, T.N. (1990). Letter to the Editor: *Cancer Res.*, **50**, 1667.

DEAR, T.N., MCDONALD, D.A. & KEFFORD, R.F. (1989). Transcriptional down-regulation of a rat gene, WDNM2, in metastatic DMBA-8 cells. *Cancer Res.*, **49**, 5323.

DEAR, T.N., RAMSHAW, I.A. & KEFFORD, R.F. (1988). Differential expression of novel gene, WDNM1, in non metastatic rat mammary adenocarcinoma cells. *Cancer Res.*, **48**, 5203.

DENHARDT, D.T., GREENBERG, A.H., EGAN, S.E., HAMILTON, R.E. & WRIGHT, J.A. (1987). Cysteine proteinase cathepsin L expression correlates closely with the metastatic potential of H-ras-transformed murine fibroblasts. *Oncogene*, **2**, 55.

EBRALIDZE, A., TULCHINSKY, E., GRIGORIAN, M. & 4 others (1989). Isolation and characterisation of a gene specifically expressed in different metastatic cells and whose deduced gene product has a high degree of homology to a $Ca^{2+}$-binding family. *Genes & Develop.*, **3**, 1086.

EGAN, S.E., MCCLARTY, G.A., JAROLIM, L. & 4 others (1987*a*) Expression of H-*ras* correlates with metastatic potential: evidence for direct regulation of the metastatic phenotype in 10T1/2 and N1H3T3 cells. *Mol. Cell. Biol.*, **7**, 830.

EGAN, S.E., WRIGHT, J.A., JAROLIM, L., YANAGIHARA, K., BASSIN, R.H. & GREENBERG, A.H. (1987*b*). Transformation by oncogenes encoding protein kinases induces the metastatic phenotype. *Science*, **238**, 202.

ELVIN, P., KERR, I.B., MCARDLE, C.S. & BIRNIE, G.D. (1988). Isolation and preliminary characteristion of cDNA clones representing mRNA's associated with tumour progression and metastasis in colorectal cancers. *Br. J. Cancer*, **57**, 36.

FEARON, E.R. & VOGELSTEIN, B. (1990). A genetic model for colorectal tumorigenesis. *Cell*, **61**, 759.

FEARON, E.R., CHO, K.R., NIGRO, J.M. & 8 others (1990). Identification of a chromosome 18q gene that is altered in colorectal cancers. *Science*, **247**, 49.

FIDLER, I.J. & KRIPKE, M.L. (1977). Metastasis results from pre-existing variant cells within a malignant tumour. *Science*, **197**, 893.

GARBISA, S., POZZATTI, R., MUSCHEL, R.J. & 5 others (1987). Secretion of type IV collagenolytic protease and metastatic phenotype; induction by transfection with c-Ha-*ras* but not c-Ha-*ras* plus Ad2-Ela. *Cancer Res.*, **47**, 1523.

GREIG, R.G., KOESTLER, T.P., TRAINER, D.L. & 6 others (1985). Tumorigenic and metastatic properties of 'normal' and ras-transfected N1H3T3 cells. *Proc. Natl Acad. Sci. USA*, **82**, 3698.

HANAHAN, D. (1988). Dissecting multistep tumorigenesis in transgenic mice. *Annul. Rev. Genet.*, **22**, 479.

HART, I.R., GOODE, N.T. & WILSON, R.E. (1989). Molecular aspects of the metastatic cascade. *Biochem. Biophys. Acta.*, **989**, 65.

HEDRICK, S.M., COHEN, D.I. & NIELSEN, E.A. (1984). Isolation of cDNA clones encoding T cell specific membrane associated protein. *Nature*, **308**, 149.

HICKS, N.J., WARD, R.V. & REYNOLDS, J.J. (1984). A fibrosarcoma model derived from mouse embryo cells. Growth properties and secretion of collagenase and metalloproteinase inhibitor (TIMP) by tumour cell lines. *Int. J. Cancer*, **33**, 835.

JOHNSON, J.P., LEHMANN, J.M., STADE, B.G., ROTHBACHER, U., SERS, C. & RIETHMULLER, G. (1989). Functional aspects of three molecules associated with metastasis development in human malignant melanoma. *Invasion Metastasis*, **9**, 338.

KERBEL, R.S. (1989). Towards an understanding of the molecular basis of the metastatic phenotype. *Invasion Metastasis*, **9**, 329.

KERBEL, R.S., WAGHORNE, C., KORCZAK, B., LAGARDE, A. & BREITMAN, M.L. (1988). Clonal dominance of primary tumours by metastatic cells: genetic analysis and biological implications. *Cancer Surveys*, **7**, 597.

KHOKHA, R., WATERHOUSE, P., YAGEL, S., OVERALL, C.M., NORTON, G. & DENHARDT, D.T. (1989). Antisense RNA-induced reduction in murine TIMP levels confers oncogenicity on Swiss 3T3 cells. *Science*, **243**, 947.

KLEIN, G. & KLEIN, E. (1985). Evolution of tumours and the impact of molecular oncology. *Nature*, **315**, 190.

KOHNE, D.E., LEVINSON, S.A. & BYERS, M.J. (1977). Room temperature method for increasing the rate of DNA reassociation by many thousand-fold. The phenol emulsion reassociation technique. *Biochemistry*, **16**, 5329.

KYPRIANOU, N. & ISAACS, J.T. (1990). Relationship between metastatic ability and H-*ras* oncogene expression in rat mammary cancer cells transfected with v-H-*ras* oncogene. *Cancer Res.*, **50**, 1449.

LEHMANN, J.M., RIETHMULLER, G. & JOHNSON, J.P. (1989). MUC 18, a marker of tumour progression in human melanoma, shows sequence similarity to the neural cell adhesion molecules of the immunoglobulin superfamily. *Proc. Natl Acad. Sci. USA*, **86**, 9891.

LIOTTA, L.A. (1988). Growth autonomy: the only requirement for metastasis? *J. Natl Can. Inst.*, **5**, 300.

MATRISIAN, L.M., BOWDEN, G.T., KRIEG, P. & 4 others (1986). The mRNA coding for the secreted protease transin is expressed more abundantly in malignant than in benign tumors. *Proc. Natl Acad. Sci. USA*, **83**, 9413.

MUSCHEL, R.J., WILLIAMS, J.E., LOWY, D.R. & LIOTTA, L.A. (1985). Harvey ras induction of metastatic potential depends upon oncogene activation and type of recipient cell. *Am. J. Pathol.*, **121**, 1.

NICOLSON, G.L. (1988). Cancer metastasis: tumor cell and host organ properties important in metastasis to specific secondary sites. *Biochem. Biophys. Acta*, **948**, 175.

NOWELL, P.C. (1976). The clonal evolution of tumor cell populations. *Science*, **194**, 23.

PHILLIPS, S.M., BENDALL, A.J. & RAMSHAW, I.A. (1990). Isolation of a gene associated with high metastatic potential in rat mammary adenocarcinomas. *J. Natl Can. Inst.*, **82**, 199.

POSTE, G. & FIDLER, I.J. (1980). The pathogenesis of cancer metastasis. *Nature*, **242**, 148.

RADLER-POHL, A., POHL, J. & SCHIRRMACHER, V. (1988). Selective enhancement of metastatic capacity in mouse bladder carcinoma cells after transfection with DNA from liver metastases of human colon carcinoma. *Int. J. Cancer*, **41**, 840.

RICH, B.E. & STEITZ, J.A. (1987). Human acidic ribosomal phosphoprotein P0, P1 and P2: analysis of cDNA clones, *in vitro* synthesis and assembly. *Mol. Cell. Biol.*, **7**, 4065.

ROSENGARD, A.M., KRUTZSH, H.C., SHEARN, A. & 6 others (1989). Reduced Nm23/Awd protein in tumour metastasis and aberrant *Drosophila* development. *Nature*, **342**, 177.

SCHALKEN, J.A., EBELING, S.B., ISAACS, J.T. & 4 others (1988). Down-modulation of fibronectin messenger RNA in metastasizing rat prostatic cancer cells revealed by differential hybridisation analysis. *Cancer Res.*, **48**, 2042.

SHARP, M.G.F., ADAMS, S.M., ELVIN, P., WALKER, R.A., BRAMMAR, W.J. & VARLEY, J.M. (1990). A sequence previously identified as metastasis-related encodes an acidic ribosomal phosphoprotein, P2. *Br. J. Cancer*, **61**, 83.

SOBEL, M.E. (1990). Metastasis suppressor genes. *J. Natl Can. Inst.*, **82**, 267.

STEEG, P.S. & LIOTTA, L.A. (1990). Reduced nm23 expression in tumor metastasis. *Proc. Am. Assoc. Cancer Res.*, **31**, 504.

STEEG, P.S., BEVILACQUA, G., KOPPER, L. & 5 others (1988). Evidence for a novel gene associated with low tumor metastatic potential. *J. Natl Cancer Inst.*, **80**, 200.

THORGEIRSSON, U.P., TURPEENIENI-HIJANEN, T., WILLIAMS, J.E. & 4 others (1985). N1H3T3 cells transfected with human tumor DNA containing activated ras oncogenes express the metastatic phenotype in nude mice. *Mol. Cell. Biol.*, **5**, 259.

TREIGER, B. & ISAACS, J.T. (1988). Expression of transfected v-Harvey-*ras* oncogene in a Dunning rat prostate adenocarcinoma and the development of high metastatic ability. *J. Urol.*, **140**, 1580.

TULCHINSKY, E.M., GRIGORIAN, M.S, EBRALIDZE, A.K., MILSHINA, N.I. & LUKANIDIN, E.M. (1990). Structure of gene *mts* 1, transcribed in metastatic mouse tumor cells. *Gene*, **87**, 219.

WALLET, V., MUTZEL, R., TROLL, H. & 5 others (1990). *Dictyostelium* nucleoside diphosphate kinase highly homologous to Nm23 and Awd proteins involved in mammalian tumor metastasis and *Drosophila* development. *J. Natl Cancer Inst.*, **82**, 1199.


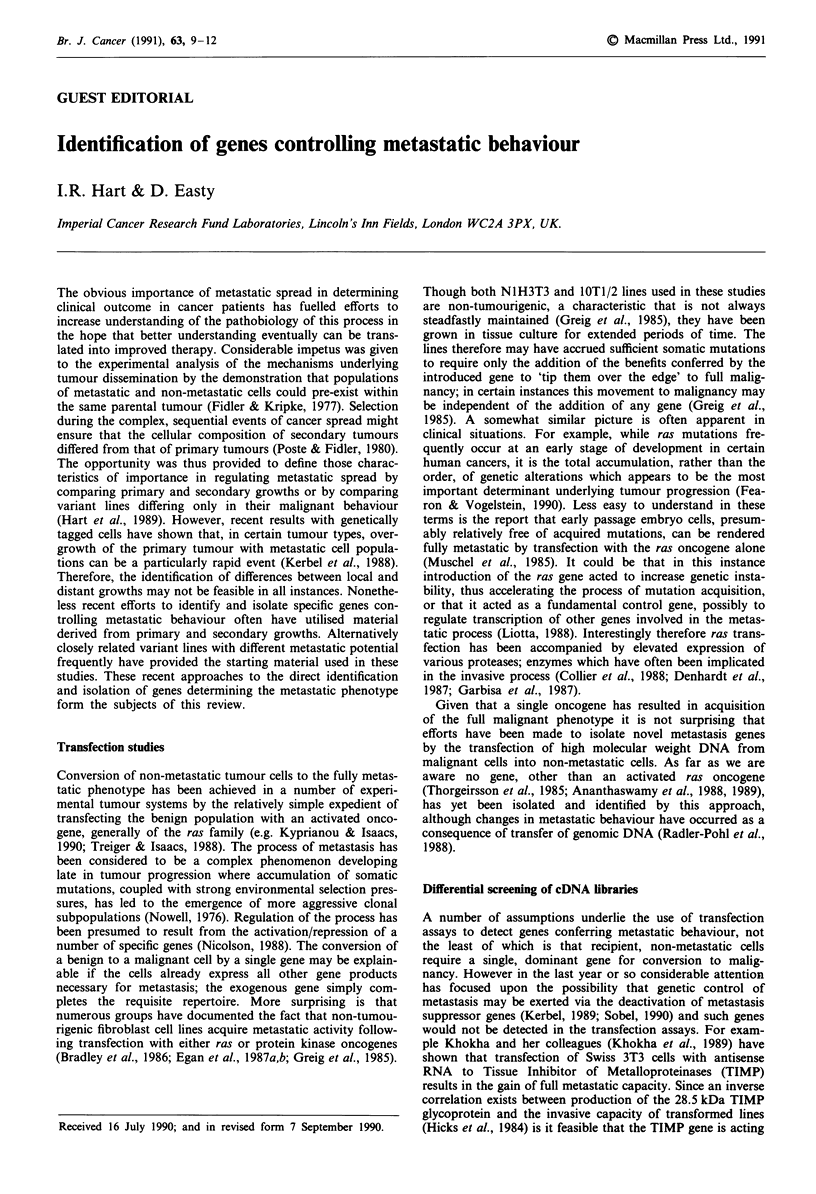

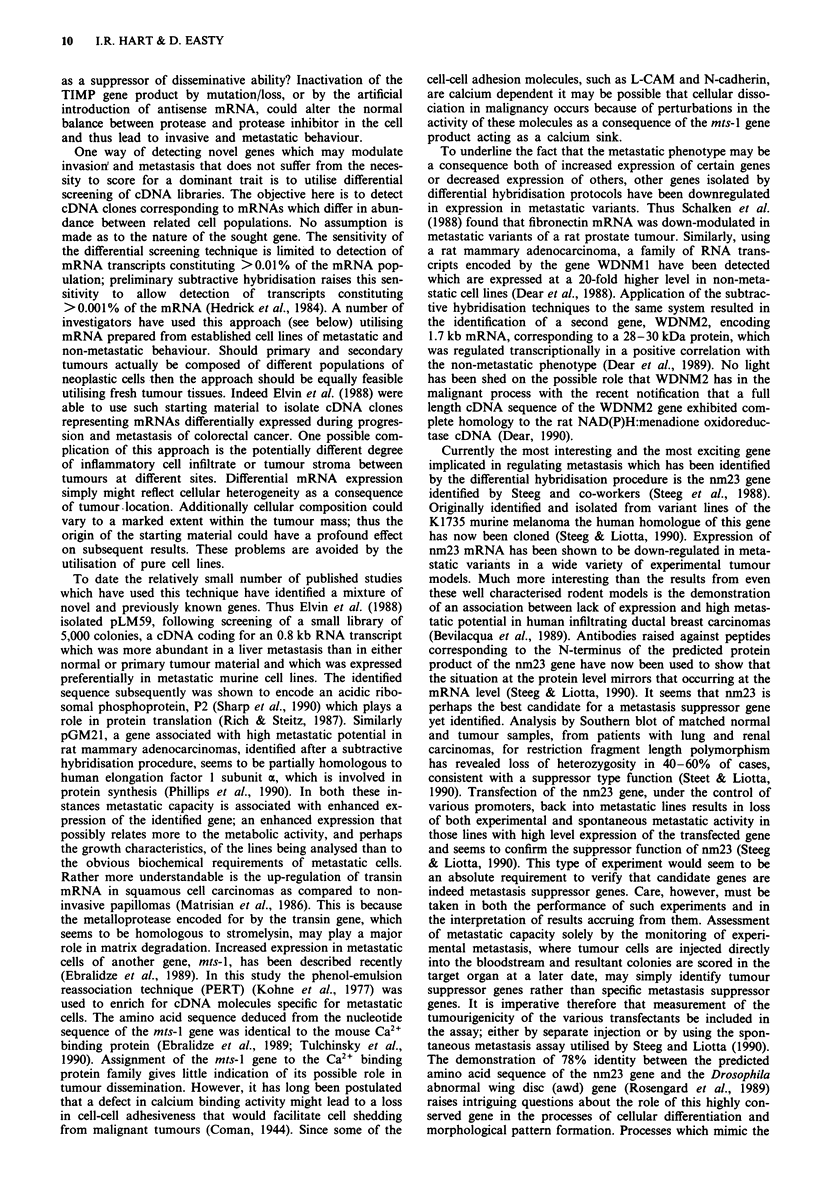

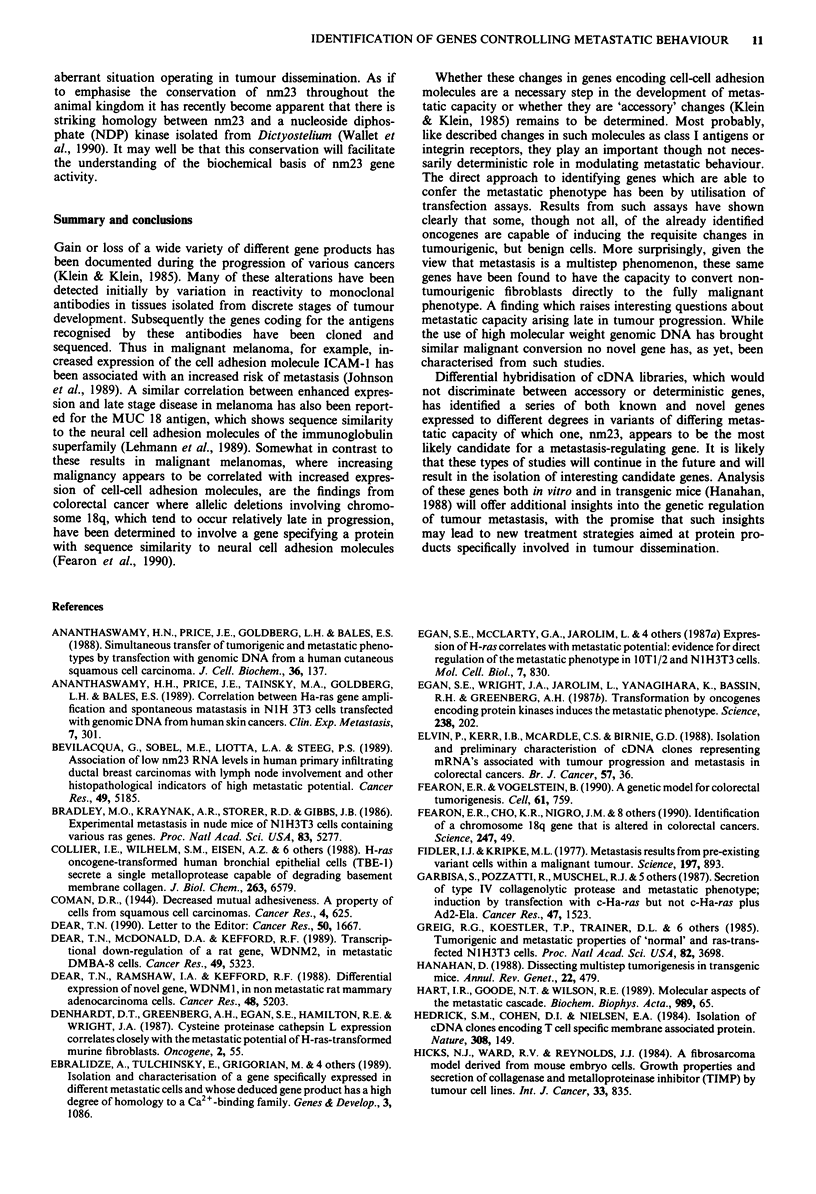

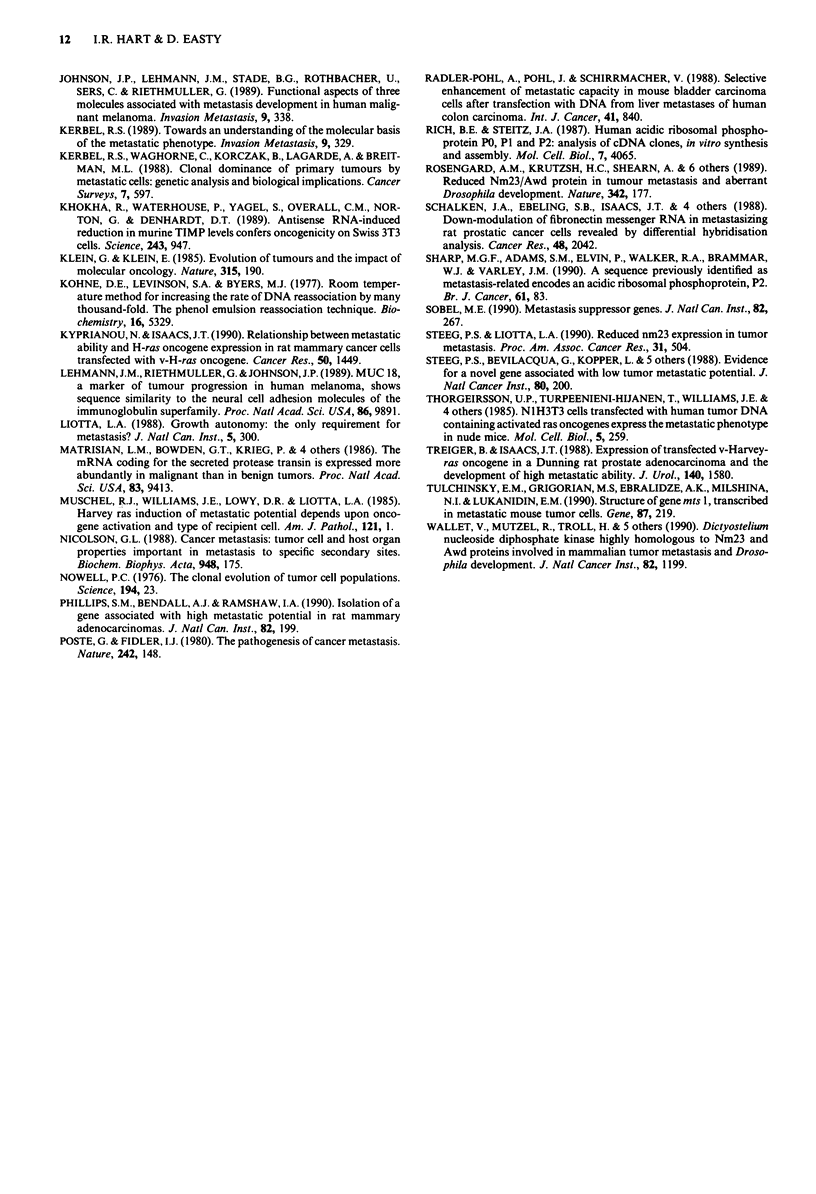


## References

[OCR_00369] Ananthaswamy H. N., Price J. E., Goldberg L. H., Bales E. S. (1988). Simultaneous transfer of tumorigenic and metastatic phenotypes by transfection with genomic DNA from a human cutaneous squamous cell carcinoma.. J Cell Biochem.

[OCR_00375] Ananthaswamy H. N., Price J. E., Tainsky M. A., Goldberg L. H., Bales E. S. (1989). Correlation between Ha-ras gene amplification and spontaneous metastasis in NIH 3T3 cells transfected with genomic DNA from human skin cancers.. Clin Exp Metastasis.

[OCR_00382] Bevilacqua G., Sobel M. E., Liotta L. A., Steeg P. S. (1989). Association of low nm23 RNA levels in human primary infiltrating ductal breast carcinomas with lymph node involvement and other histopathological indicators of high metastatic potential.. Cancer Res.

[OCR_00389] Bradley M. O., Kraynak A. R., Storer R. D., Gibbs J. B. (1986). Experimental metastasis in nude mice of NIH 3T3 cells containing various ras genes.. Proc Natl Acad Sci U S A.

[OCR_00394] Collier I. E., Wilhelm S. M., Eisen A. Z., Marmer B. L., Grant G. A., Seltzer J. L., Kronberger A., He C. S., Bauer E. A., Goldberg G. I. (1988). H-ras oncogene-transformed human bronchial epithelial cells (TBE-1) secrete a single metalloprotease capable of degrading basement membrane collagen.. J Biol Chem.

[OCR_00406] Dear T. N., McDonald D. A., Kefford R. F. (1989). Transcriptional down-regulation of a rat gene, WDNM2, in metastatic DMBA-8 cells.. Cancer Res.

[OCR_00411] Dear T. N., Ramshaw I. A., Kefford R. F. (1988). Differential expression of a novel gene, WDNM1, in nonmetastatic rat mammary adenocarcinoma cells.. Cancer Res.

[OCR_00404] Dear T. N. (1990). Re: T. Neil Dear et al., Transcriptional down-regulation of a rat gene, WDNM2, in metastatic DMBA-8 cells. Cancer Res., 49: 5323-5328, 1989.. Cancer Res.

[OCR_00416] Denhardt D. T., Greenberg A. H., Egan S. E., Hamilton R. T., Wright J. A. (1987). Cysteine proteinase cathepsin L expression correlates closely with the metastatic potential of H-ras-transformed murine fibroblasts.. Oncogene.

[OCR_00422] Ebralidze A., Tulchinsky E., Grigorian M., Afanasyeva A., Senin V., Revazova E., Lukanidin E. (1989). Isolation and characterization of a gene specifically expressed in different metastatic cells and whose deduced gene product has a high degree of homology to a Ca2+-binding protein family.. Genes Dev.

[OCR_00429] Egan S. E., McClarty G. A., Jarolim L., Wright J. A., Spiro I., Hager G., Greenberg A. H. (1987). Expression of H-ras correlates with metastatic potential: evidence for direct regulation of the metastatic phenotype in 10T1/2 and NIH 3T3 cells.. Mol Cell Biol.

[OCR_00435] Egan S. E., Wright J. A., Jarolim L., Yanagihara K., Bassin R. H., Greenberg A. H. (1987). Transformation by oncogenes encoding protein kinases induces the metastatic phenotype.. Science.

[OCR_00441] Elvin P., Kerr I. B., McArdle C. S., Birnie G. D. (1988). Isolation and preliminary characterisation of cDNA clones representing mRNAs associated with tumour progression and metastasis in colorectal cancer.. Br J Cancer.

[OCR_00447] Fearon E. R., Vogelstein B. (1990). A genetic model for colorectal tumorigenesis.. Cell.

[OCR_00456] Fidler I. J., Kripke M. L. (1977). Metastasis results from preexisting variant cells within a malignant tumor.. Science.

[OCR_00460] Garbisa S., Pozzatti R., Muschel R. J., Saffiotti U., Ballin M., Goldfarb R. H., Khoury G., Liotta L. A. (1987). Secretion of type IV collagenolytic protease and metastatic phenotype: induction by transfection with c-Ha-ras but not c-Ha-ras plus Ad2-E1a.. Cancer Res.

[OCR_00466] Greig R. G., Koestler T. P., Trainer D. L., Corwin S. P., Miles L., Kline T., Sweet R., Yokoyama S., Poste G. (1985). Tumorigenic and metastatic properties of "normal" and ras-transfected NIH/3T3 cells.. Proc Natl Acad Sci U S A.

[OCR_00471] Hanahan D. (1988). Dissecting multistep tumorigenesis in transgenic mice.. Annu Rev Genet.

[OCR_00475] Hart I. R., Goode N. T., Wilson R. E. (1989). Molecular aspects of the metastatic cascade.. Biochim Biophys Acta.

[OCR_00479] Hedrick S. M., Cohen D. I., Nielsen E. A., Davis M. M. (1984). Isolation of cDNA clones encoding T cell-specific membrane-associated proteins.. Nature.

[OCR_00484] Hicks N. J., Ward R. V., Reynolds J. J. (1984). A fibrosarcoma model derived from mouse embryo cells: growth properties and secretion of collagenase and metalloproteinase inhibitor (TIMP) by tumour cell lines.. Int J Cancer.

[OCR_00492] Johnson J. P., Lehmann J. M., Stade B. G., Rothbächer U., Sers C., Riethmüller G. (1989). Functional aspects of three molecules associated with metastasis development in human malignant melanoma.. Invasion Metastasis.

[OCR_00498] Kerbel R. S. (1989). Towards an understanding of the molecular basis of the metastatic phenotype.. Invasion Metastasis.

[OCR_00504] Kerbel R. S., Waghorne C., Korczak B., Lagarde A., Breitman M. L. (1988). Clonal dominance of primary tumours by metastatic cells: genetic analysis and biological implications.. Cancer Surv.

[OCR_00510] Khokha R., Waterhouse P., Yagel S., Lala P. K., Overall C. M., Norton G., Denhardt D. T. (1989). Antisense RNA-induced reduction in murine TIMP levels confers oncogenicity on Swiss 3T3 cells.. Science.

[OCR_00514] Klein G., Klein E. (1985). Evolution of tumours and the impact of molecular oncology.. Nature.

[OCR_00518] Kohne D. E., Levison S. A., Byers M. J. (1977). Room temperature method for increasing the rate of DNA reassociation by many thousandfold: the phenol emulsion reassociation technique.. Biochemistry.

[OCR_00524] Kyprianou N., Isaacs J. T. (1990). Relationship between metastatic ability and H-ras oncogene expression in rat mammary cancer cells transfected with the v-H-ras oncogene.. Cancer Res.

[OCR_00529] Lehmann J. M., Riethmüller G., Johnson J. P. (1989). MUC18, a marker of tumor progression in human melanoma, shows sequence similarity to the neural cell adhesion molecules of the immunoglobulin superfamily.. Proc Natl Acad Sci U S A.

[OCR_00534] Liotta L. A. (1988). Growth autonomy: the only requirement for metastasis?. J Natl Cancer Inst.

[OCR_00538] Matrisian L. M., Bowden G. T., Krieg P., Fürstenberger G., Briand J. P., Leroy P., Breathnach R. (1986). The mRNA coding for the secreted protease transin is expressed more abundantly in malignant than in benign tumors.. Proc Natl Acad Sci U S A.

[OCR_00544] Muschel R. J., Williams J. E., Lowy D. R., Liotta L. A. (1985). Harvey ras induction of metastatic potential depends upon oncogene activation and the type of recipient cell.. Am J Pathol.

[OCR_00549] Nicolson G. L. (1988). Cancer metastasis: tumor cell and host organ properties important in metastasis to specific secondary sites.. Biochim Biophys Acta.

[OCR_00554] Nowell P. C. (1976). The clonal evolution of tumor cell populations.. Science.

[OCR_00558] Phillips S. M., Bendall A. J., Ramshaw I. A. (1990). Isolation of gene associated with high metastatic potential in rat mammary adenocarcinomas.. J Natl Cancer Inst.

[OCR_00567] Radler-Pohl A., Pohl J., Schirrmacher V. (1988). Selective enhancement of metastatic capacity in mouse bladder carcinoma cells after transfection with DNA from liver metastases of human colon carcinoma.. Int J Cancer.

[OCR_00573] Rich B. E., Steitz J. A. (1987). Human acidic ribosomal phosphoproteins P0, P1, and P2: analysis of cDNA clones, in vitro synthesis, and assembly.. Mol Cell Biol.

[OCR_00578] Rosengard A. M., Krutzsch H. C., Shearn A., Biggs J. R., Barker E., Margulies I. M., King C. R., Liotta L. A., Steeg P. S. (1989). Reduced Nm23/Awd protein in tumour metastasis and aberrant Drosophila development.. Nature.

[OCR_00583] Schalken J. A., Ebeling S. B., Isaacs J. T., Treiger B., Bussemakers M. J., de Jong M. E., Van de Ven W. J. (1988). Down modulation of fibronectin messenger RNA in metastasizing rat prostatic cancer cells revealed by differential hybridization analysis.. Cancer Res.

[OCR_00589] Sharp M. G., Adams S. M., Elvin P., Walker R. A., Brammar W. J., Varley J. M. (1990). A sequence previously identified as metastasis-related encodes an acidic ribosomal phosphoprotein, P2.. Br J Cancer.

[OCR_00595] Sobel M. E. (1990). Metastasis suppressor genes.. J Natl Cancer Inst.

[OCR_00603] Steeg P. S., Bevilacqua G., Kopper L., Thorgeirsson U. P., Talmadge J. E., Liotta L. A., Sobel M. E. (1988). Evidence for a novel gene associated with low tumor metastatic potential.. J Natl Cancer Inst.

[OCR_00608] Thorgeirsson U. P., Turpeenniemi-Hujanen T., Williams J. E., Westin E. H., Heilman C. A., Talmadge J. E., Liotta L. A. (1985). NIH/3T3 cells transfected with human tumor DNA containing activated ras oncogenes express the metastatic phenotype in nude mice.. Mol Cell Biol.

[OCR_00614] Treiger B., Isaacs J. (1988). Expression of a transfected v-Harvey-ras oncogene in a Dunning rat prostate adenocarcinoma and the development of high metastatic ability.. J Urol.

[OCR_00619] Tulchinsky E. M., Grigorian M. S., Ebralidze A. K., Milshina N. I., Lukanidin E. M. (1990). Structure of gene mts1, transcribed in metastatic mouse tumor cells.. Gene.

[OCR_00624] Wallet V., Mutzel R., Troll H., Barzu O., Wurster B., Veron M., Lacombe M. L. (1990). Dictyostelium nucleoside diphosphate kinase highly homologous to Nm23 and Awd proteins involved in mammalian tumor metastasis and Drosophila development.. J Natl Cancer Inst.

